# The Regenerating Gene I**α** Is Overexpressed in Atrophic Gastritis Rats with Hypergastrinemia

**DOI:** 10.1155/2011/403956

**Published:** 2011-09-22

**Authors:** Shujie Chen, Jing Zhong, Qunyan Zhou, Xiaofeng Lu, Liangjing Wang, Jianmin Si

**Affiliations:** ^1^Laboratory of Digestive Disease, Sir Run Run Shaw Clinical Medicine Institution of Zhejiang University, Hangzhou 310003, China; ^2^Department of Gastroenterology, Second Affiliated Hospital of Zhejiang University School of Medicine, Hangzhou 310009, China

## Abstract

The role of gastrin on the development of atrophic gastritis (AG) and its relationship with the expression of RegI*α* *in vivo* remain unclear. We established experimental AG in rats by combination administration with sodium salicylate, alcohol, and deoxycholate sodium. The mean score of inflammation in gastric antrum in AG rats was significantly elevated (*P* < 0.05), while the number of glands dramatically decreased (*P* < 0.05). In addition, the cell proliferation in gastric glands was increased in experimental AG rats, as determined by immunohistochemistry staining of PCNA and GS II. The level of serum gastrin in AG rats was significantly elevated relative to that of normal rats (*P* < 0.01). Moreover, the expression of RegI*α* protein and its receptor mRNA was increased in gastric tissues in AG rats (*P* < 0.05). Taken together, we demonstrated that the overexpression of Regl*α* is related with hypergastrinemia in AG rats.

## 1. Introduction

Atrophic gastritis (AG) was defined as the loss of glands and/or replacement by intestinal glands in gastric mucosa, which has been recognized as initial step in the process of AG-dysplasia—gastric cancer (intestinal type) consequence [1, 2]. AG is classified as two major types, autoimmune atrophic gastritis and multifocal atrophic gastritis, and the later disease involves both the antrum and corpus of stomach and represents an increased risk for gastric cancer [1, 3]. Among multiple regulators, growing evidences indicated that growth factors may play an important role in the progression from chronic AG to gastric cancer [4]. The polypeptide hormone gastrin has been demonstrated to be an essential growth factor in gastric carcinogenesis [5]. In corpus-associated gastric atrophy, the maintenance of G cells and the loss of parietal cells could lead to hypergastrinaemia [5]. In contrast, in antrum-predominant AG, though the reduction of G cells inhibits the release of gastrin [6], the increase of inflammation in antrum mucosa induces gastric gland atrophy, intestinal metaplasia, and even tumorigenesis [7]. However, the roles of gastrin in the development of AG are not fully understood.

The regenerating gene (Reg) I*α* was originally isolated from regenerating pancreatic islet cells [8]. In the stomach, RegI*α* is expressed in the enterochromaffin-like (ECL) cells in response to water immersion stress-induced gastric mucosa damage [9–12]. It has been revealed that gastrin stimulates the ECL cells proliferation in *Helicobacter pylori* (*H. pylori*-)associated gastritis. Studies also demonstrated that gastrin and *H. pylori* could stimulate the expression of RegI*α* through binding to its distinct promoter elements [13]. Unexpectedly, evidence showed that gastrin could not directly promote the proliferation of cultured rat gastric epithelial cells, and it was proposed that this effect may be indirectly mediated through RegI*α*. We have shown that exogenous administration of gastrin-17 (G17) peptide could promote AGS gastric cancer cells proliferation and upregulate RegI*α* expression [14], but whether the gastrin is associated with the expression of RegI*α* *in vivo* remains unclear. Studies showed that RegI*α* is overexpressed in *H. pylori*-induced gastritis and gastric ulcers [15, 16]. Our recent works revealed that RegI*α* expression was involved in progression from active gastritis and precancerous lesions to gastric cancer [14]. Studies have also demonstrated that RegI*α* promoted gastric cell growth and differentiation in the neck zone, suggesting a role as a potent trophic agent of progenitor cells of the gastric fundic mucosa [17]. Therefore, study on roles of RegI*α* in AG animal model may provide further insight into the relationship between them.

In the present study, we successfully established animal model of AG with hypergastrinemia and showed that the expression of RegI*α* is related with the level of gastrin. Our results may provide evidence that RegI*α* could be a potential therapeutic target of AG with hypergastrinemia.

## 2. Material and Methods

### 2.1. Establishment of Atrophic Gastritis Model in Rats

Twenty male Wistar rats (130–150 g) were obtained from Shanghai Slac Laboratory Animal Co. Ltd. (Shanghai, China). The study was in compliance with the Declaration of Helsinki. Atrophic gastritis in rats was established according to our previously published methods [18]. Briefly, rats (*n* = 10) were intragastric administered, with mixing 2% sodium salicylate and 30% alcohol, and 20 mmol/L deoxycholate sodium for 10 weeks, and deprived of water by replacement with 0.1% ammonia water. In addition, control group rats (*n* = 10) were administrated with same amount of PBS. Rats were placed in stainless cages with 5 animals in each group, at temperature (22 ± 2)°C, humidity 55% ~ 65%, with 12 hours dark and light cycles.

### 2.2. Gastric Tissues Preparation

At the ending of modeling experiments, animals were sacrificed. The glossy appearance including color, plica, and mucin in gastric mucosa was observed after cutting the stomach along the lesser and greater curvature. Then, the gastric specimens were immediately immersed in 10% buffered formalin and embedded in paraffin. Paraffin sections (5 *μ*m) were routinely stained with hematoxylin stain. Remaining specimens were frozen in liquid nitrogen for further analysis.

### 2.3. Histological Assessment

Histological change in gastric antrum was assessed by the diagnostic criteria of gastritis in Houston in 1996 [19, 20]. The mean number of infiltrated inflammatory cells was calculated in each of 10 microscopic fields of antrum mucosa and ranked as 4 score system (0 = normal; 1 = few inflammatory cells infiltration in pit or basal region of gastric glands; 2 = moderate number of inflammatory cells infiltration, which localized within two thirds of gastric glands; 3 = large amount of inflammatory cells infiltration into whole gastric glands). The number of gastric glands per area was randomly observed in 5 microscopic fields.

### 2.4. Immunohistochemistry Stain

Expression of proliferating cell nuclear antigen (PCNA) (1 : 1,000, Dako Company, Denmark) and GSII (plant lectin Griffonia Simplicifolia, 1 : 2,500; Vector Laboratories, USA) in rat gastric glands was detected using immunohistochemistry stains according to the manufacturer's instructions. Biotin-conjugated GSII, which recognizes N-acetyl-D-glucosamine (GlcNAc) at the end of O-glycosylated sugar chains, was used to estimate the proliferation zone in the neck regions of gastric glands.

### 2.5. ELISA Assay

The blood samples were drawn from the rat femoral artery. Gastrin levels (ng/mL) in serum were measured by using a rat gastrin ELISA kit (R&D, USA) according to the manufacturer's instructions.

### 2.6. Quantitative Real-Time PCR

Total RNA was extracted with Trizol (Invitrogen, USA) according to the protocol. RNA (1 *μ*g) was reverse transcribed into cDNA using Oligo (dT) 15 primers and TaKaRa reverse transcriptase (TaKaRa, China). Quantitative real-time PCR was performed using the SYBR Green Master Mix Kit (TaKaRa, China) in an ABI 7500 machine. Primers of RegI receptor (RegIR) were (forward) 5′-ACAAGGTAGTGGTGGTGTGGAACTC-3′; (reverse) 5′-TGTCTCTATCTCATTCCAGGGCAAG-3′. The expression levels of RegIR mRNA were determined using the 2^−ΔΔCT^ method. GAPDH was used as an internal control. The relative fold change of RegIR mRNA in AG group was compared with which of normal group, which was normalized as a reference value of 1.0.

### 2.7. Western Blot Analysis

Total protein from gastric tissues was extracted with radio immunoprecipitation assay (RIPA) lysis buffer containing protease inhibitors. 40 *μ*g of total protein were loaded in each well, and the proteins were separated by 12% SDS-PAGE and blotted onto polyvinylidene difluoride (PVDF) membranes. The membranes were incubated with rabbit polyclonal anti-mouse RegI (1 : 500, Lifespan BioSciences, USA) overnight at 4°C. Horseradish peroxidase-conjugated goat anti-rabbit IgG (1 : 2,500, MultiSciences Biotech, China) was used for enhanced chemiluminescent detection with an LAS-4000 image system.

### 2.8. Statistical Analysis

Student's *t*-test was performed to compare with two-independent data, while two-tailed Chi-square or Fisher's exact tests were used for comparison of categorical variables. A cutoff of *P* < 0.05 was applied for statistical significance.

## 3. Results

### 3.1. Pathological Findings in Rats with Atrophic Gastritis

**Figure 1:**
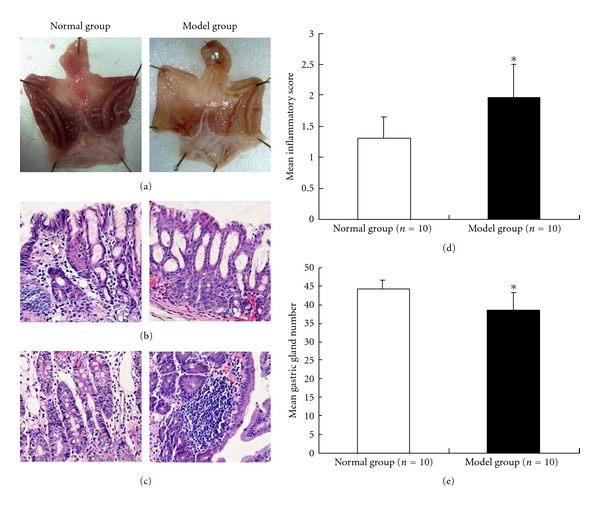
Pathological finding in rats with atrophic gastritis. (a) Gloss appearance of gastric mucosa. Flat with pale appearance and thin mucin on the gastric mucosa in atrophic gastritis group rats (right diagram) when compared with normal rats (left diagram). (b) H&E (×100) staining of gastric gland. Irregular arrangement and cystic dilation of gastric glands in atrophic gastritis (right diagram). (c) H&E (×200) staining showed that neutrophils and lymphocytes infiltrated into the gastric glands in rats with atrophic gastritis (right diagram). (d) The mean number of infiltrated inflammatory cells was calculated in each of 10 microscopic fields of gastric antrum glands. The mean inflammation score (mean ± s.d) was 1.95 ± 0.55 and 1.3 ± 0.34, respectively, in atrophic gastritis and normal rats. (e) The number of gastric glands in each of 1 mm area (mean ± SD) was randomly analyzed in 5 microscopic fields, which was 39.8 ± 4.59 and 44.2 ± 2.57, respectively, in atrophic gastritis and normal rats. Normal group: normal rats; Model group: atrophic gastritis rats. *P < 0.05.

We observed that the glossy gastric mucosa is flat or disappeared, with pale appearance and thin mucin in rats with experimental atrophic gastritis (Figure 1(a)). As shown in Figures 1(b) and 1(c), irregular arrangement and multiple cystic dilation in gastric glands, as well as massive neutrophil and lymphatic cell infiltration between gastric glands, were found by light microscope in atrophic gastritis rats. There are no detectable histological intestinal metaplasia and H. pylori infection in gastric mucosa from both group rats.

We observed that the mean score of inflammation (1.95 ± 0.55) in atrophic gastritis group was significantly elevated when compared to that of 1.3 ± 0.34 in control group (*P* < 0.05) (Figure 1(d)), while the number of glands in per area of gastric antrum was significantly decreased in atrophic gastritis group relative to normal groups (39.8 ± 4.59 versus 44.2 ± 2.57, *P* < 0.05). These data indicated that the experimental atrophic gastritis in rats was successfully established.

### 3.2. Cell Proliferation Is Increased in Atrophic Gastritis Rats

**Figure 2:**
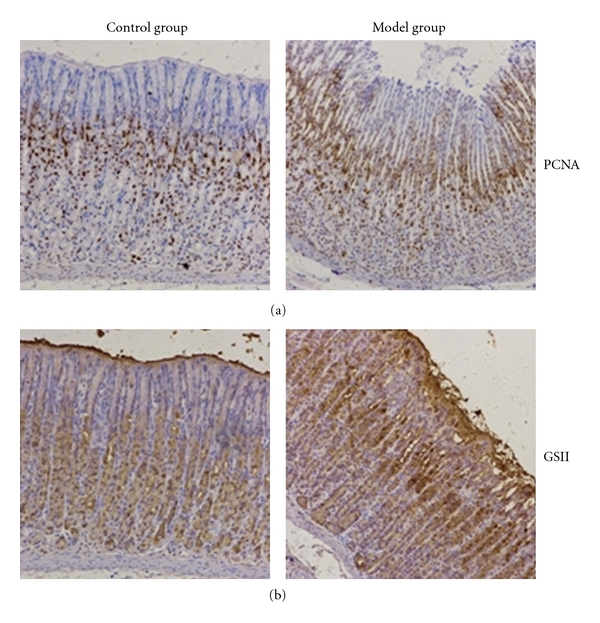
Immunohistochemistry stains of PCNA and GSII in gastric glands. (a) Positive stain of PCNA in cellular nucleus, which are mainly localized in deep layer of gastric glands in normal rats (left diagram), while extended to whole glands in atrophic gastritis (right diagram). (b) GSII was moderately positive stained in the neck and deep layer of gastric glands in normal rats (left diagram). It showed of strong stain in the neck and upper areas in the gastric glands in rats with atrophic gastritis (right diagram). Control group: normal control rats; Model group: atrophic gastritis rats. *P < 0.05, **P < 0.01.

To assess the cell proliferation in gastric glands of atrophic gastritis rats, we performed immunohistochemistry stains of PCNA and GS II in gastric tissues. Our results demonstrated that PCNA was positively stained in cellular nucleus of gastric glands, particular in the neck and deep layer areas. The areas of PCNA stain were obviously enlarged in atrophic gastritis glands relative to normal stomach (Figure 2(a)). Moreover, GS II, another cell proliferation marker which secreted from gastric mucosa neck cells, was mainly localized in the neck area of gastric glands in normal rats. In atrophic gastritis rats, the GS II was extensively and strongly stained in neck area as well as upper layer epithelial cells (Figure 2(b)). The neck areas of gastric gland were usually considered as where the progenitor cells localized. Our results revealed that cell proliferation in gastric glands was increased in rats with experimental atrophic gastritis, as determined by two of biomarkers, PCNA and GS II.

### 3.3. Hypergastrinemia in Rats with Autrum-Predominant Atrophic Gastritis

**Figure 3:**
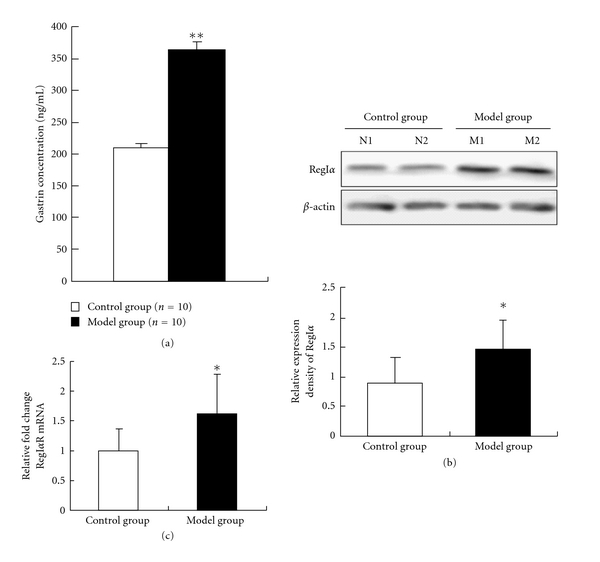
The RegI*α* protein and its receptor mRNA were overexpressed in atrophic gastritis rats with hypergastrinemia. (a) The average serum gastrin levels (ng/mL) in atrophic gastritis and normal rats were determined by ELISA assay. (b) Representative results of RegI*α* protein expression in gastric tissues from atrophic gastritis and normal rats (upper lane) were performed by immunoblot experiments. Band densities were quantified and protein levels were normalized to *β*-actin (lower diagram). (c) The expression of RegI*α* receptor mRNA was measured by quantity real-time PCR and calculated using the value of 2^−ΔΔCT^. Relative fold change in atrophic gastritis group was compared with that of normal group, which was normalized as a reference value of 1.0. Control group: normal control rats; Model group: atrophic gastritis rats. *P < 0.05, **P < 0.01.

Our previous data showed that administration of gastrin could stimulate cell proliferation *in vitro* [14]. To confirm whether the level of gastrin was induced by the proliferative gastric glands, we detected the concentration of gastrin in rats with atrophic gastritis as well as in normal rats. ELISA assays found that the level of serum gastrin was dramatically increased to 375.0 ± 103.9 ng/mL in atrophic gastritis rats with comparison of 209.6 ± 48.2 ng/mL in normal rats (*P* < 0.01) (Figure 3(a)). Our data indicated that rats with autrum-predominant atrophic gastritis have hypergastrinemia.

### 3.4. RegI*α* and Its Receptor RegIR Are Overexpressed in Experimental Rats with Atrophic Gastritis

To determine the possible role of RegI*α* protein, an important downstream regulator of gastrin, in the pathogenesis of atrophic gastritis, we examine the expression of RegI*α* and its receptor RegIR in rats. As shown in Figure 3(b), Western blot detected that the expression of total RegI*α* protein from atrophic gastritis glands is relatively higher than those from normal control rats (relative RegI*α* expression density 1.45 ± 0.54 versus 0.87 ± 0.44, *P* < 0.05). In addition, the quantity real-time PCR analysis demonstrated that the expression level of RegI*α* receptor mRNA in atrophic gastritis group was upregulated to about 1.6-fold relative to normal control group (Figure 3(c)). Our results showed that RegI*α* overexpression may be associated with hypergastrinemia in a rat model of atrophic gastritis.

## 4. Discussion

Multiple pathological factors are involved in the process of AG, which could continuously damage the defense barrier in gastric mucosa and lead to the loss of glands [3]. *H. pylori*, bile refluxes, and alcohol intake were considered as the main stimulators of AG. We simulated the above possible factors by combination administration with ammonia water, sodium salicylate, deoxycholate sodium, and alcohol and successfully established the antrum-predominant AG in rats. Furthermore, the rats with AG had hypergastrinemia, and pathological findings are matched with the diagnostic criteria according to the Houston International Gastritis Classification [19, 20]. Many recent studies have suggested that patients with *H. pylori* infection have higher serum gastrin levels [21, 22]. *H. pylori* infection may produce hyperammonia and change the status of low pH value in the antrum of stomach. The feedback by reduction of gastric acid could lead to hypergastrinemia. Another possible explanation of hypergastrinemia in our rats model with AG is that the secretion of gastrin from G cells in antrum was increased with chemically inducible inflammation [5]. 

We also demonstrated that there are abnormal patterns of cell proliferation in gastric gland in experimental AG rats by immunohistochemistry stain assays. The proliferating cell nuclear antigen (PCNA) found in the nucleus encodes for a protein that is involved in cellular DNA synthesis and cell cycle progression [23]. The expression of PCNA is a common marker associated with cell proliferation [24]. The lectin *Griffonia simplicifolia* II (GSII) is secreted from gastric mucosa neck cell and specifically reacted with nonreducing terminal N-acetyl glucosamine (GlcNAc) [25, 26]. We found that the combination stains levels with PCNA and GS II were significantly higher in gastric tissues from AG rats. The results indicated that the increase of abnormal cell proliferation exists in the neck zone of gastric glands in AG rats.

Recently, studies showed that RegI protein is a possible downstream mediator of gastrin-induced gastric mucosa regeneration. RegI could directly stimulate the proliferation of gastric mucosa cells generated from rats, while gastrin did not have this effect [27]. Lansoprazole medication inducible hypergastrinemia may increase the thickness of mucosa and the RegI expression in rat stomachs. The induction of Reg protein by hypergastrinemia was abolished by treatment with gastrin receptor antagonist AG-041R in rats [27]. Further studies also suggested that gastrin stimulation of ECL cells had a growth-promoting effect by enhancing the production of Reg protein [27, 28]. In addition, RegI might play an important role in mediating the effects of gastrin on the proliferation and differentiation of ECL cells in cell culture systems [5, 28]. We revealed that the higher expression of RegI*α* was presented in AG rats with hypergastrinemia. Our previous observations showed that exogenous administration of gastrin (G17) promotes RegI*α* expression in AGS cells [14]. Taken together, our results provided additional evidence that the expression of RegI is related with the level of gastrin. 

However, the pathway for gastrin-mediated RegI*α* regulation has not yet been fully understood. Interestingly, gastrin and *H. pylori* could upregulate the expression of luciferase reporters transfected with RegI promoter (−2111 bp and −104 bp) in primary mouse gastric glands, which suggested that gastrin could regulate the transcriptional induction of RegI*α* [13]. We have reported that *β*-catenin was accumulated and translocated to the nucleus when AGS cells were cultured with exogenous gastrin [14]. *REGIα* and *REGIIIα* genes were considered as possible downstream targets of the Wnt/*β*-catenin pathway during liver tumorigenesis *in vitro* [29]. So we proposed that gastrin stimulation of RegI might be via *β*-catenin pathways. 

In summary, we demonstrated that RegI*α* overexpression is related with hypergastrinemia in atrophic gastritis rats.

## References

[1] Genta RM (1998). Atrophy and atrophic gastritis: one step beyond the Sydney system. *Italian Journal of Gastroenterology and Hepatology*.

[2] Correa P (1988). Chronic gastritis: a clinico-pathological classification. *American Journal of Gastroenterology*.

[3] Fox JG, Wang TC (2007). Inflammation, atrophy, and gastric cancer. *Journal of Clinical Investigation*.

[4] Dockray GJ, Varro A, Dimaline R, Wang T (2001). The gastrins: their production and biological activities. *Annual Review of Physiology*.

[5] Watson SA, Grabowska AM, El-Zaatari M, Takhar A (2006). Gastrin-active participant or bystander in gastric carcinogenesis?. *Nature Reviews Cancer*.

[6] Wang LJ, Chen SJ, Chen Z, Cai JT, Si JM (2006). Morphological and pathologic changes of experimental chronic atrophic gastritis (CAG) and the regulating mechanism of protein expression in rats. *Journal of Zhejiang University Science B*.

[7] Zavros Y, Eaton KA, Kang W (2005). Chronic gastritis in the hypochlorhydric gastrin-deficient mouse progresses to adenocarcinoma. *Oncogene*.

[8] Terazono K, Yamamoto H, Takasawa S (1988). A novel gene activated in regenerating islets. *Journal of Biological Chemistry*.

[9] Asahara M, Mushiake S, Shimada S (1996). Reg gene expression is increased in rat gastric enterochromaffin-like cells following water immersion stress. *Gastroenterology*.

[10] Ashcroft FJ, Varro A, Dimaline R, Dockray GJ (2004). Control of expression of the lectin-like protein Reg-1 by gastrin: role of the Rho family GTPase RhoA and a C-rich promoter element. *Biochemical Journal*.

[11] Fukui H, Kinoshita Y, Maekawa T (1998). Regenerating gene protein may mediate gastric mucosal proliferation induced by hypergastrinemia in rats. *Gastroenterology*.

[12] Kazumori H, Ishihara S, Hoshino E (2000). Neutrophil chemoattractant 2*β* regulates expression of the Reg gene in injured gastric mucosa in rats. *Gastroenterology*.

[13] Steele IA, Dimaline R, Pritchard DM (2007). Helicobacter and gastrin stimulate Reg1 expression in gastric epithelial cells through distinct promoter elements. *American Journal of Physiology*.

[14] Zhou Q, Lu X, Gan L (2010). Role of REG I*α* in gastric carcinogenesis: gastrin-associated proliferative and anti-apoptotic activities. *Molecular Medicine Reports*.

[15] Fukui H, Fujii S, Takeda J (2004). Expression of Reg I*α* protein in human gastric cancers. *Digestion*.

[16] Sekikawa A, Fukui H, Fujii S (2005). Possible role of REG I*α* protein in ulcerative colitis and colitic cancer. *Gut*.

[17] Kinoshita Y, Ishihara S, Kadowaki Y, Fukui H, Chiba T (2004). Reg protein is a unique growth factor of gastric mucosal cells. *Journal of Gastroenterology*.

[18] Wang LJ, Zhou QY, Chen Y (2009). Muscovite reverses gastric gland atrophy and intestinal metaplasia by promoting cell proliferation in rats with atrophic gastritis. *Digestion*.

[19] Dixon MF, Genta RM, Yardley JH, Correa P Classification and grading of gastritis. The updated Sydney System.

[20] Dixon MF, Genta RM, Yardley JH, Correa P (1996). Classification and grading of gastritis: the updated Sydney system. *American Journal of Surgical Pathology*.

[21] Chuang CH, Sheu BS, Yang HB (2004). Hypergastrinemia after Helicobacter pylori infection is associated with bacterial load and related inflammation of the oxyntic corpus mucosa. *Journal of Gastroenterology and Hepatology*.

[22] Peach HG, Barnett NE (2000). Determinants of basal plasma gastrin levels in the general population. *Journal of Gastroenterology and Hepatology*.

[23] Rizzo MG, Ottavio L, Travali S (1990). The promoter of the human proliferating cell nuclear antigen (PCNA) gene is bidirectional. *Experimental Cell Research*.

[24] Leung AY, Leung JC, Chan LY (2005). Proliferating cell nuclear antigen (PCNA) as a proliferative marker during embryonic and adult zebrafish hematopoiesis. *Histochemistry and Cell Biology*.

[25] Kang W, Rathinavelu S, Samuelson LC, Merchant JL (2005). Interferon gamma induction of gastric mucous neck cell hypertrophy. *Laboratory Investigation*.

[26] Sawaguchi A, Tojo H, Kawano JI, Okamoto M, Suganuma T (2001). Immunocytochemical demonstration of the secretory dynamics of zymogenic contents in rat gastric gland processed by high-pressure freezing/freeze substitution, with special references to phospholipase A(2) and phospholipase C*γ*1. *Histochemistry and Cell Biology*.

[27] Chiba T, Fukui H, Kinoshita Y (2000). Reg protein: a possible mediator of gastrin-induced mucosal cell growth. *Journal of Gastroenterology*.

[28] Miyaoka Y, Kadowaki Y, Ishihara S (2004). Transgenic overexpression of Reg protein caused gastric cell proliferation and differentiation along parietal cell and chief cell lineages. *Oncogene*.

[29] Cavard C, Terris B, Grimber G (2006). Overexpression of regenerating islet-derived 1 alpha and 3 alpha genes in human primary liver tumors with *β*-catenin mutations. *Oncogene*.

